# A 3D‐Printed Hybrid Nasal Cartilage with Functional Electronic Olfaction

**DOI:** 10.1002/advs.201901878

**Published:** 2020-01-10

**Authors:** Yasamin A. Jodat, Kiavash Kiaee, Daniel Vela Jarquin, Rosakaren Ludivina De la Garza Hernández, Ting Wang, Sudeep Joshi, Zahra Rezaei, Bruna Alice Gomes de Melo, David Ge, Manu S. Mannoor, Su Ryon Shin

**Affiliations:** ^1^ Division of Engineering in Medicine Department of Medicine Harvard Medical School Brigham and Women's Hospital Cambridge MA 02139 USA; ^2^ Department of Mechanical Engineering Stevens Institute of Technology Hoboken NJ 07030 USA; ^3^ Instituto Tecnológico y de Estudios Superiores de Monterrey Calle del Puente #222 Col. Ejidos de Huipulco, Tlalpan C.P. 14380 México D.F. Mexico; ^4^ Instituto Tecnológico y de Estudios Superiores de Monterrey Av. Eugenio Garza Sada 2501 Sur, Tecnológico 64849 Monterrey N.L. Mexico; ^5^ School of Medicine Jiangsu University Zhenjiang Jiangsu 212013 China; ^6^ Department of Chemical and Petroleum Engineering Sharif University of Technology Azadi Ave 11365‐11155 Tehran Iran; ^7^ Department of Engineering of Materials and Bioprocesses School of Chemical Engineering University of Campinas Campinas São Paulo 13083‐852 Brazil

**Keywords:** 3D bioprinting, bioelectronic noses, biomaterials, bionic organs, electrochemical biosensors

## Abstract

Advances in biomanufacturing techniques have opened the doors to recapitulate human sensory organs such as the nose and ear in vitro with adequate levels of functionality. Such advancements have enabled simultaneous targeting of two challenges in engineered sensory organs, especially the nose: i) mechanically robust reconstruction of the nasal cartilage with high precision and ii) replication of the nose functionality: odor perception. Hybrid nasal organs can be equipped with remarkable capabilities such as augmented olfactory perception. Herein, a proof‐of‐concept for an odor‐perceptive nose‐like hybrid, which is composed of a mechanically robust cartilage‐like construct and a biocompatible biosensing platform, is proposed. Specifically, 3D cartilage‐like tissue constructs are created by multi‐material 3D bioprinting using mechanically tunable chondrocyte‐laden bioinks. In addition, by optimizing the composition of stiff and soft bioinks in macro‐scale printed constructs, the competence of this system in providing improved viability and recapitulation of chondrocyte cell behavior in mechanically robust 3D constructs is demonstrated. Furthermore, the engineered cartilage‐like tissue construct is integrated with an electrochemical biosensing system to bring functional olfactory sensations toward multiple specific airway disease biomarkers, explosives, and toxins under biocompatible conditions. Proposed hybrid constructs can lay the groundwork for functional bionic interfaces and humanoid cyborgs.

## Graphical abstract

An odor‐perceptive nose‐like hybrid is developed using multi‐material 3D bioprinting. The mechanically robust cartilage‐mimetic tissue is integrated with a biosensing platform tunable to “sniff out” a wide range of chemical structures, such as explosives. This integrative approach lays the groundwork for functional bionic interfaces and humanoid cyborgs.

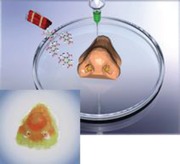

## 1 Introduction

The rapid growth of the biomedical engineering field in the past few decades has enabled the emergence of bionic organs, a highly serviceable asset for replicating specific organ functions and increasing the accuracy of in vitro test models, with the aim to eventually replace native organs. To create bionic organs, high‐precision and functional electronic devices need to be integrated into viable engineered biological systems. While the two stem from fully disparate classes of materials and fabrication techniques, recent research has been focused on closing this gap by introducing techniques and protocols optimized for co‐fabrication of biocompatible and implantable bionic organs.^1^ The current state of development of bionic organs is still impeded by several challenges, particularly in mimicking the complexity of tissue or organ structure and functionality. The intricate heterogeneity in biological structures and the physical properties of human organs has intrigued a need for multi‐material and multi‐cellular hybrid designs. Various studies have introduced a well‐established protocol to fabricate macro‐scale tissues and organ‐like constructs with resemblance to the biological structure and physical properties of the target tissue or organ.^2^ However, tissue‐specific functionalities such as sensory and auditory capabilities, olfaction, and vision are often chiefly missing from the replicas.

Among the various sensory organs in the body, the nose has a great potential to be targeted for bionic organ engineering, as both the tissue and function can be mimicked through tissue engineering techniques and odor‐detecting electronic devices. The nasal cartilage tissue is majorly composed of hyaline cartilage, which consists of densely packed collagen and proteoglycan‐based extracellular matrix (ECM) embedded with chondrocytes.^3^ Moreover, it has a relatively simple structure along with mechanically robust and elastic properties compared to other tissues in the body.^4^ These attributes facilitate engineering 3D nasal cartilage tissues using a combination of biomaterials and microfabrication techniques. On the other hand, the sensing and olfactory system in this organ is endowed with intricate and unique physical and biological properties. With the ability to discriminate thousands of volatile compounds and chemical structures, the olfactory system plays an important role in assisting humans with perception of the outer environment. Through studying the chemical structure of the olfactory receptors (ORs) and their function, the specific binding of each of these proteins to odorant compounds with similar chemical structures can be achieved, paving the way for engineering an odor‐perceptible artificial nose.

Two distinct research communities have dedicated their efforts to nose organ reconstruction. In the first group, where the focus is on emulating the function of the nose via electronic nose sensor devices, the biological features and physical functions of a native nose are neglected.^5^ As such, interfacing the electronic sensing system with the tissue is also ignored. The electronic noses often consist of a sensing unit, which is immobilized with odor‐specific receptors which capture the target molecule and transduce the binding event to an electrical signal. Sensing mechanisms such as field effect transistors (FETs), quartz crystal microbalance, and electrochemical (EC) sensor units have proved successful.^6^ Electronic noses have been developed for a range of applications to detect toxic gases,^7^ food quality monitoring^8^ as well as being used as a diagnostic tool for diseases such as pneumonia^9^ and lung cancer.^10^ An obstructive factor in integrating the commonly developed sensor devices with tissues is pertained to the harsh conditions in which the sensor devices operate. Moreover, selection of toxic chemicals (e.g., specific electrolytes) and electrode materials to improve the sensing capability can create a noncytocompatible environment for most tissues.^11^ The second community, on the other hand, has focused merely on modification or reconstruction of the nasal anatomy for cosmetic purposes, or for after the organ has been physically damaged (e.g., those caused by traumatic accidents). Given the structural features and replication feasibilities of the nasal organ, engineering and development of cartilage‐mimicking tissues has proved practically successful in several studies.^12^ Additionally, some groups in this category have targeted cartilage regeneration and tissue repair.[qv: 12c,13] The elaborate structure of the nasal passageway plays an integral part in conditioning air for olfaction and respiration. This conditioning includes precise controlling of the gaseous fluidic mechanics of the inhaled air and adjustments to humidity and temperature, and filtering and air flow characteristics such as flow rate, intermittence, and regime (laminar, transitional, or turbulent).^14^ Moreover, this structure allows the airflow velocity to be decreased near the olfactory epithelium, facilitating full contact between gas molecules and the lined surface of the olfactory epithelium thus increasing the sensitivity of odor detection.[qv: 14c,15] To achieve enhanced artificial olfaction with close relevance to the native structure, such anatomical elaborations should be integrated into the bioelectronic noses.

Here, we aim to bridge this gap between the two communities to create an effective biocompatible functional hybrid nose‐like tissue construct. Previously, a 3D printing–based approach was pursued to bring auditory functionality to auricular cartilage via developing a 3D‐printed bionic ear.^16^ Following a similar strategy, we are targeting the retention of olfaction to develop a hybrid nasal tissue. To the best of our knowledge, there have not been any attempts toward the creation of a hybrid nasal tissue with the capability of odor sensing and olfactory system retention. Specifically, we have developed a chondrocyte‐laden 3D‐bioprinted cartilage‐like construct with an electronic olfaction‐mimetic biosensor. Employing 3D bioprinting technology is especially beneficial in this approach as it allows for the creation of 3D hybrid platforms with desired geometries and high precision. While 3D printing parameters can be readily engineered to fit the cellular environment requirements, the structure can be coupled with an electronic functional element to create a viable hybrid device.^16^, ^17^ Moreover, photo‐crosslinkable hydrogel‐based bioinks composed of gelatin methacryloyl (GelMA) and polyethylene glycol dimethacrylate (PEGDMA) can form a mechanically robust and biocompatible 3D microenvironment to support cartilage cell growth and their differentiation by tuning the mechanical properties of the bioinks. As such, two distinct bioinks with stiff and soft mechanical properties composed of different hydrogel concentrations could be optimized to mimic the mechanical properties of the native ECM of nasal cartilage to allow for nasal cartilage tissue formation.

To integrate olfaction (biosensing based on OR immobilization) and odor sensing (e.g., biosensing using peptides and aptamers) into the 3D‐printed cartilage tissue constructs while preserving biological culture conditions, EC‐based biosensing presents an applicable choice with advantages such as high sensitivity, label‐free detection, a wide linear detection range, and excellent detection limit. Additionally, EC‐based biosensors possess flexibility in detection ability; for instance, the sensor can be conveniently functionalized with odorant receptor proteins to detect a wide range of human‐detectable odors and chemicals. Furthermore, scalability and ease of miniaturization of the EC‐based biosensors on various substrates (e.g., glass, paper, flexible polymeric substrates, etc.) facilitate their integration into live tissues and organs. In this paper, the biosensing mechanism is based on label‐free EC impedance spectroscopy (EIS) to further improve sensitivity and circumvent acute cytotoxicity issues induced through labeling odorant receptors with electrochemically active specific molecules. Nonetheless, common electrolytes, such as potassium ferricyanide (K_3_Fe(CN)_6_), are often toxic to cellular environments and affect cell‐laden system viability. To operate the biosensor under biological culture conditions, we employ a culture media‐based electrolyte system with a weak and nearly negligible EC redox activity, lower conductivity compared to K_3_Fe(CN)_6_, but adequate sensitivity to pico‐level concentrations of the analyte.^18^ Developing such cytocompatible biosensors can open a pathway toward augmented sensing in hybrid organs; for instance, it is possible to functionalize the system with airborne pathogenic biomarkers,^19^ volatile disease metabolites,^20^ or odorless contaminants,^21^ and thus achieve a cyborg olfactory organ. Such a bionic nose can prove useful in applications such as the detection of explosives, illegal drugs, or food poisoning.

## 2 Results and Discussion

### 2.1 Mechanically Tunable Dual Bioink System

**Figure 1:**
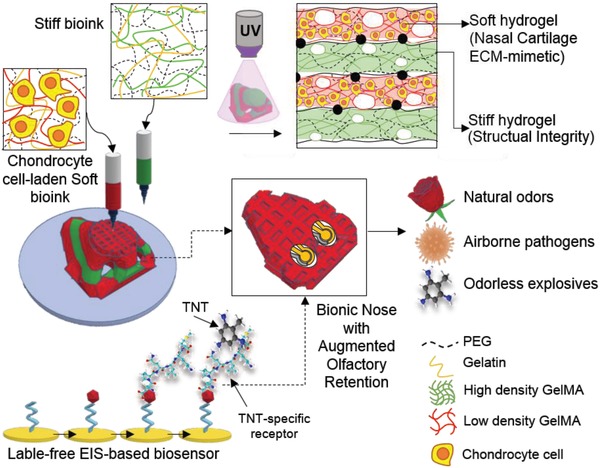
Schematic diagram showing the procedure of printing the nose. The soft ink including the chondrocyte‐laden solution and the stiff ink are both loaded in the printer. Afterward, dual printing is performed on top of the microfabricated sensor functionalized with a TNT‐specific peptide. This hybrid system can be tuned to detect a range of targets such as natural odors, airborne pathogens, and odorless explosives.

The dimensions of common self‐standing cell‐laden 3D‐printed constructs often do not exceed few centimeters. Several studies that created constructs beyond a few centimeters tall faced problems, such as losing the precision in geometry as short as a few minutes after printing, due to the swelling or low stiffness of the bioink.^22^ To overcome this challenge, one solution is to increase the viscosity or stiffness of the bioink to enhance the printability and reduce swelling.^23^ However, in many cases, increasing the bioink stiffness results in reduced cell viability and proliferation imposed by the physical constraints of the now stiffer matrix.^24^ Consequently, the trade‐off between geometry precision and cell behaviors remains an ongoing challenge. To overcome this challenge, two distinct bioinks with stiff and soft mechanical properties were used to form a mechanically robust and biocompatible 3D‐printed construct to support cartilage cell growth and differentiation within desired geometries. To enable two distinct mechanical properties within a single printed construct, we employed a multi‐material 3D bioprinting technique to create a self‐standing 3D hybrid platform with desired stiffness and geometries (>1.5 cm height of printed constructs). **Figure** **1** shows the procedure for fabrication of the hybrid nasal cartilage using a dual nozzle printing system. In the current study, a combination of GelMA and PEGDMA was employed to create a viable scaffold supportive of cartilage regeneration while being capable of maintaining the geometry of the structure even weeks after culture. High hydrophilicity, biocompatibility, and considerably low immunogenicity are some of the features of the mentioned polymers.^25^ Owing to the reversible thermal gelation of GelMA pre‐polymer solutions, the viscosity of the bioinks can be further reinforced prior to printing through physical gelation. Post‐printing chemical crosslinking can be achieved through photo‐crosslinking with UV light.

Two bioinks consisting of different compositions of PEGDMA and GelMA were selected and denoted as “soft” and “stiff,” with the latter containing higher concentrations of each material. The former promotes cell adhesion and creates a suitable stiffness for chondrocyte growth while the latter creates a structural scaffold to retain the shape and higher stiffness for mechanical strength. Following pre‐polymer solution preparation, the soft ink was mixed with the cells to create a cell‐laden bioink, while the stiff ink was prepared and printed in the original acellular conditions. Addition of sacrificial biomaterials, such as gelatin, to the pre‐polymer solution of both inks allowed for improved printability and a geometrically defined structure post‐printing. Later the gelatin was dissolved from printed constructs during cell culture processes. To develop two different soft and stiff bioinks, we first tested the printability of bioinks with various combinations of gelatin, GelMA, and PEGDMA, reaching good printability. At the same time, we fabricated bulk hydrogels and then measured the mechanical properties (data not shown). We then selected two different compositions of soft and stiff bioinks: 5% GelMA/5% PEGDMA/6.5% gelatin and 8% GelMA/10% PEGDMA/3% gelatin, respectively. We confirmed two distinct levels of mechanical stiffness for crosslinked soft and stiff bulk hydrogels as 20.3 ± 1.1 and 67.3 ± 1.1 kPa, respectively (Figure S1a, Supporting Information). The fracture point of stiff bulk gel was found to be 125 kPa at 50% strain, five times higher than the soft bulk gel. In addition, the microstructures of soft and stiff crosslinked bioinks were observed by scanning electron microscopy (SEM), and were found to be micro‐porous structures. However, while the soft gel tended to easily collapse during the freeze‐drying process due to its weak mechanical stiffness, the stiff gel was able to maintain its micro‐porosity (Figure S1b,c, Supporting Information).

**Figure 2:**
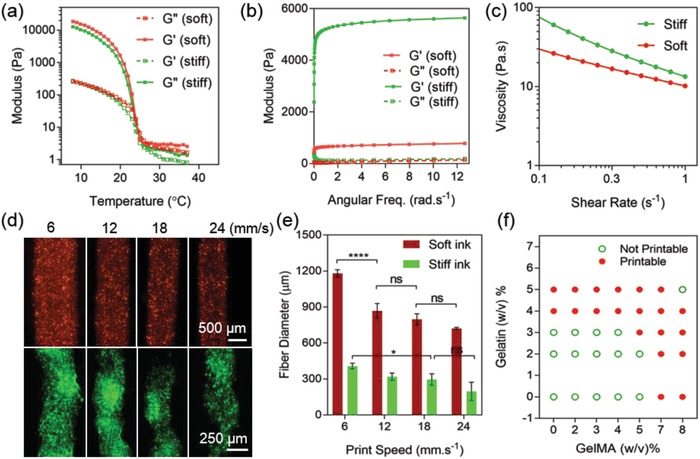
Rheological properties of the soft and stiff inks. a) Temperature‐dependent behavior of soft and stiff inks affecting the storage (*G*') and loss (*G*”) moduli, thus printability of each ink (*N* = 3). b) Storage (*G*') and loss (*G*”) moduli of soft and stiff inks across a frequency sweep (*N* = 3). c) Change of viscosity with increments of shear stress for both inks showing the shear thinning behavior of the inks (*N* = 3). d) Fluorescence imaging of dyed extruded fibers using the soft (red) and stiff (green) bioinks across a variation of nozzle printing speeds. The soft ink creates thicker fibers due to lower viscosity. e) Characterization of fiber diameter by nozzle printing speed for the extruded fibers shown in (d). The extruded fiber diameters ranged from 200 to 480 µm for the stiff ink and 550 to 1200 µm for the soft ink with lower viscosity (*N* = 4). f) Printability of the GelMA and gelatin optimized for printing.

To understand and characterize the temperature‐sensitive and fluidic behavior of each ink, the physical properties of the pre‐polymer solution of soft and stiff bioinks were measured. Due to the temperature‐dependent sensitivity of the gelatin and GelMA pre‐polymer solutions, the viscosity of the inks was directly influenced by temperature, resulting in the creation of straight fibers and a more precise geometry under 19 °C and highly reduced printability above 27 °C (**Figure** **2**a). Therefore, the incubation time for each ink required careful optimization to reach suitable printable viscosity between 17 and 22 °C. Frequency sweep measurements showed that both soft and stiff bioinks possessed appropriate rheological properties to be used in bioprinting. The higher storage modulus (*G*' > *G*”) across the range of frequencies studied indicated gel‐like behavior, and therefore, the bioinks' suitability to hold the shape of the bioprinted structures, especially for the stiff ink, in which *G*' was nearly sevenfold greater than that of the soft ink (Figure 2b). Moreover, both inks presented a non‐Newtonian shear‐thinning behavior, with the power law index (*n*) being < 1. Such an index value is favorable for bioprinting due to the capacity of the material in behaving as a fluid under high shear stress, allowing a smoother nozzle extrusion, and behaving as a rigid body under low shear, after bioprinting (Figure 2c and Table S1, Supporting Information).

Due to the lower viscosity of the soft ink, the extruded fiber was thicker compared to the stiff ink at the same printing speed. By adjusting the moving speed of the nozzle, the diameter of the extruded lines was optimized to reach similar printability in both inks (Figure 2d,e). Speeds higher than 24 mm s^−1^ resulted in breakage in lines and thus loss of printability. Therefore, we chose 18 mm s^−1^ for the soft ink nozzle and 6 mm s^−1^ to print the stiff layers. The air pump pressure was kept constant around 30 kPa for both inks. The diameter of the extruded fiber using stiff ink ranged from 200 to 480 µm while the soft ink with lower viscosity exhibited a higher extruded fiber diameter range (≈550 to ≈1200 µm). As such, the diameters of the extruded fibers in the mentioned optimized nozzle speeds were found to be around 800 µm for the soft layers and 400 µm for the stiff layers. Moreover, by adjusting the gelatin to GelMA ratio in ink compositions, the printability of the inks was optimized to create continuous fibers without needle blockage (Figure 2f).

**Figure 3:**
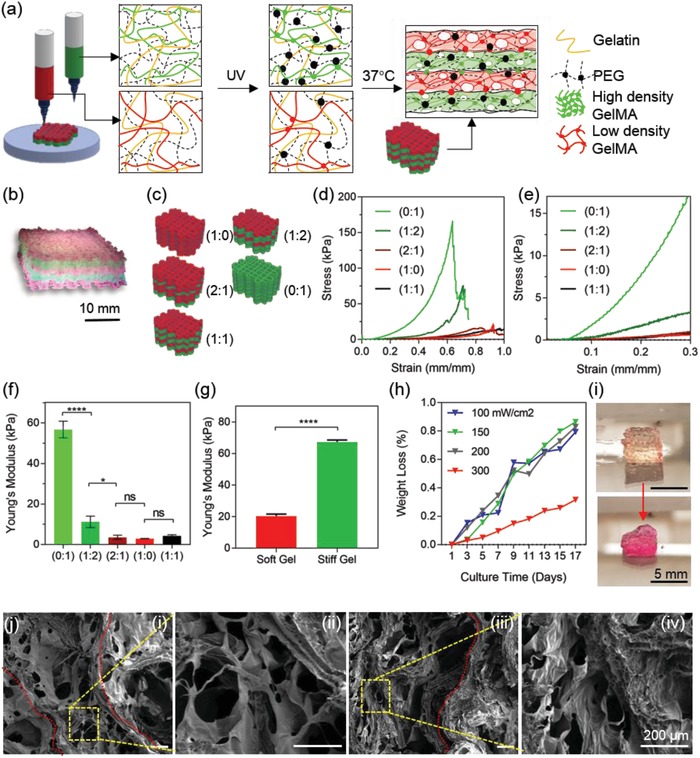
a) Schematic of each ink composition after the UV crosslinking procedure and 37 °C incubation. The dots represent crosslinked sites upon UV exposure. The stiff inks include more crosslinked sites due to higher GelMA concentration. After incubation at 37 °C, the gelatin is dissolved, leaving the construct with a porous structure. b) A cube printed with soft (red) and stiff (green) inks (1:1) and crosslinked at 150 mW cm^−2^. c) Different ratios of soft (red) to stiff (green) were selected and tested. (2:0) and (0:2) represent the softest and stiffest constructs, respectively. d,e) Stress–strain curve of the different print ratio composites depicted in (c). f) Young's modulus of the printed composite constructs (*N* = 4). g) Young's modulus for soft and stiff bulk gels (nonprinted). h) Percentage of weight loss in the hydrogel samples during the 17 day culture. The UV crosslinking intensity was optimized to reduce the degradation rate and 300 mW cm^−2^ represents the least degradation. i) One of the cubes crosslinked at 100 mW cm^−2^ before (top) and after (bottom) degradation within the 17 day culture. Scale bar is 5 mm. j) SEM imaging of printed soft (i,ii) and stiff (iii,iv) gels. Red line indicates the printed fiber edge. Scale bar is 200 µm.

Since the cell‐laden layers require delivery of oxygen and nutrients through the thick printed construct, the permeability of the constructs had to be adjusted to allow for the circulation of nutrients and oxygen. We modified the printing parameters such as 3D slicing and infill ratio of the geometry to create a macro‐porous structure without compromising the shape integrity after printing and during culture. Using a 15–20% infill ratio in slicing the 3D model into layers met both criteria. After optimizing the printing parameters, the PEGDMA and GelMA pre‐polymer chains in the printed constructs were crosslinked and solidified upon UV radiation. **Figure** **3**a depicts the crosslinking procedure of the bioinks. Moreover, the formation of covalent bonds between the GelMA and PEGDMA among the layers helped to avoid any lamination issues between the stiff and soft printed layers despite their dissimilar mechanical properties. As a result, a multi‐layered cube composed of soft (red) and stiff (green) printed gel layers could be obtained (Figure 3b). Comparing the 3D computer‐aided design (CAD) model dimensions with those of the 3D‐printed product, we observed a 21% increase in the area accompanied with a 3% reduction in height before swelling the printed construct in the biological media. Assuming that both the stiff/soft hydrogels of the printed construct swelled in all three dimensions and the effects of swelling and collapsing could be superposed, we can conclude that the original collapse of the gel was around 18% of the original height. This reduction could be pertained to a greater collapse in the soft layers than that of the stiff layers as a result of low stiffness and less crosslinking density of the soft hydrogel. Also, gravitational forces of thick whole constructs might lead to the closure of internal pores in the soft hydrogel.^26^ We then tested the printed constructs with various ratios of soft and stiff printed layers in terms of structural integrity and mechanical properties (Figure 3c–f). In terms of the effect of the printing process on the mechanical properties, the existence of print infill relatively reduced the stiffness of fully stiff (0:1) and fully soft (1:0) printed constructs with ≈57 and ≈3 kPa Young's modulus, respectively (Figure 3f) compared to the nonprinted bulk crosslinked gels (≈20 kPa, soft and ≈67 kPa, stiff; Figure 3g and Figure S1a, Supporting Information). This reduction happened due to the creation of larger cavities within the microgrid structure as well as the propagation of cracks on the edge of the printed lines which caused breakage. Due to the presence of infill and patterned cavities in all printed constructs, the Young's moduli of the printed constructs were not directly comparable with those of bulk gels. Therefore, an “infill factor” was introduced to all obtained Young's moduli to normalize the printed constructs by the nominal print area, actual area, and weight of the printed samples. More specifically, fully stiff (0:1) and fully soft (1:0) printed constructs were normalized by their bulk gel counterparts through calculating the approximate density of each construct using the measured weight and dimensions. Next, two infill factors were obtained for the fully soft and fully stiff hydrogels (i.e., soft infill and stiff infill). Composite infill factors for 1:1, 1:2, and 2:1 were introduced by composing different ratios of fully stiff and fully soft infill factors (e.g., 1:1 infill was made with (1X soft infill+1X stiff infill)/2).

By increasing the soft:stiff ratio, or in other words, increasing the number of stiff layers, higher geometrical accuracy and mechanical properties can be obtained. For instance, fully stiff (0:1) hydrogels showed a significant difference in Young's modulus compared to the composite hydrogels (1:0, 1:1, and 1:2). This tunability can be especially useful for 3D‐bioprinted constructs with complex printing patterns and nozzle movements in all *X‐*, *Y‐*, and *Z*‐directions, namely, those in a 3D nose model. However, increasing the overall stiffness of the constructs can inhibit the growth and ECM secretion of the cells. In this case, using higher soft:stiff ratios would provide a more appropriate substrate for chondrocyte growth. Mechanical compression tests on the samples with different soft:stiff ratios proved no significant difference between the Young's modulus of 1:0, 1:1, and 1:2 ratios. However, the Young's modulus increased significantly between 2:1 and 1:2 ratios. As a result, the (1:2) ratio was selected to create a composite construct with structural integrity while at the same time providing mechanical properties comparable to those of the ECM required for chondrocyte growth and cartilage development. Although the mechanical stiffness of the native mature nasal cartilage matrix was reported as 100–400 kPa,^27^ studies have shown greater chondrocyte maturation and high cartilage matrix diffusion in engineered tissues with lower mechanical properties (10–30 kPa) compared to the native cartilage.^16^, ^28^ For instance, a recent study by Levett et al. showed that GelMA–hyaluronic acid (HA)–chondroitin sulfate (CS) scaffolds with mechanical properties ranging from 20 to 40 kPa can induce chondrogenic differentiation and high production of cartilage ECM including collagen II (COLII) and glycosaminoglycan (GAG) content. Moreover, embedding chondrocytes in the composite GelMA‐HA‐CS scaffolds led to a significant increase in mechanical properties up to 150 kPa after 8 weeks of culture. It is hypothesized that optimizing initial conditions such as large pore sizes and proper initial mechanical properties can lead to a large matrix secretion and higher mechanical properties in the engineered cartilage‐like tissues over longer culture periods. As such, it is suggested that initially softer constructs can transform to the stiffest ECM over time more effectively than initially stiff hydrogels, since the latter can impede the formation and dispersion of the new ECM produced by the cells.^29^

Nonetheless, the degradation test of the printed construct with 1:2 ratio confirmed that structural integrity was preserved even after 17 days of culture while the final stiffness was enough to provide a suitable growing environment for the cells (Figure 3h). The percentage weight loss of the GelMA‐PEG‐gelatin acellular constructs was caused by both dissolving of gelatin and degradation of GelMA chains at 37 °C upon incubation. GelMA polypeptide chains were chemically crosslinked via conjugated methacrylate groups, however, most of the peptide bonds in the GelMA polypeptide chains could be broken by hydrolysis from salt ions existing in a water‐based solvent (ea. biological media) combined with an elevated temperature of around 40 °C.^30^ In terms of cell‐laden structures, the protein structure of the GelMA hydrogels permits cells to enzymatically degrade and remodel the gel for cell spreading and expanding in the degraded spaces.^31^ Moreover, by tuning the UV intensity, the printed construct could obtain a tunable range of degradation properties as well as maintain its shape integrity to minimize weight loss (Figure S2, Supporting Information). Increasing the crosslinking intensity resulted in a higher elastic modulus, however, exceeding 150 mW cm^−2^ led to the reduction of cell viability due to the release of free radicals as well as possible damages and mutations in the cell DNA (Figure S3, Supporting Information). The optimal UV intensity for simultaneous targeting of structural integrity and high cell viability was found to be 100 mW cm^−2^ with an exposure time of 80 s at an 8 cm distance from the light source. Figure 3i shows the degraded hydrogel constructs crosslinked at 100 mW cm^−2^ on day 1 and day 17 of culture at 37 °C. By incubating the structure at 37 °C after UV crosslinking, the gelatin was removed from the structure and dissolved in the culture media. As a result, a highly microporous structure was achieved, as confirmed by SEM imaging (Figure 3j). Interestingly, printed hydrogels maintained a relatively higher porous structure compared to bulk crosslinked gels (Figure S1b, Supporting Information). This event can be pertained to the local chemical and physical crosslinking of the extruded fibers as exposed to the printer halogen light and lower temperature of the bed, thus creating a structurally robust construct. As seen in Figure 3j‐i,ii, the printed soft layers maintained a highly porous structure with larger pore sizes compared to the stiff layers (Figure 3j‐iii,iv, thus promoting the perfusability of the constructs.

### 2.2 A Supportive Microenvironment for Chondrocyte Culture and Growth

After optimizing printing parameters, cell‐laden bioinks were characterized to study the feasibility of cell growth and functionality in the multi‐material printed structure. Chondrocytes, a representative cell type composing the nasal cartilage, were cultured and encapsulated in the soft bioink for cell‐laden bioprinting. A major challenge in expanding the primary chondrocytes in conventional 2D monolayer dishes has been the loss of chondrogenic phenotypes and the occurrence of “dedifferentiation” after a few passages. Dedifferentiation of chondrocytes is often followed by the gradual decay of chondrocyte‐specific molecular markers such as GAGs, aggrecan (ACAN), and COLII.^32^ At this stage, chondrocytes would lose the original rounded morphology and tend to a more extended fibroblast‐like morphology.^33^ Several studies have proven that “re‐differentiation” can be achieved by embedding and culturing the chondrocytes in high‐density 3D gel suspensions such as agarose,^34^ alginate beads,^35^ or 3D collagen‐based matrices.^36^ Moreover, the chondrocyte markers such as COLII deposition are shown to be restored through 3D culture. In the present study, the printing and culture of the encapsulated chondrocytes in the GelMA‐based soft bioink led to the maintenance of a typical rounded morphology in the chondrocytes (Figure S4, Supporting Information). The presence of RGD (Arg‐Gly‐Asp) binding sites in the GelMA hydrogel was previously shown to enable high cellular adhesion.^37^

**Figure 4:**
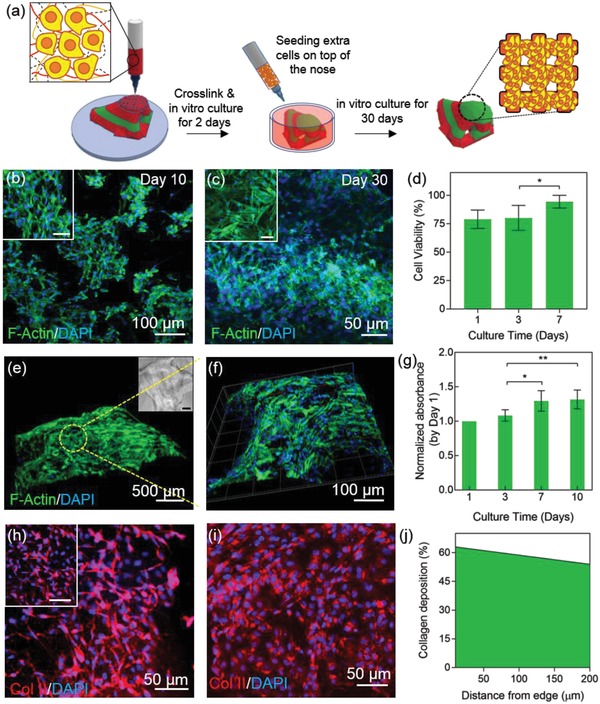
Characterization of cell‐laden ink during in vitro culture. a) Schematic diagram of cell integration in the bioprinted construct. b,c) Confocal images of 3D soft gels immunostained with F‐actin/DAPI on day 10 and day 30 of culture. The scale bars in the insets are 50 µm. d) Cell viability after printing shows a small decrease but the cells revive until day 7 (*N* = 3). e,f) 3D reconstruction of Z‐stack images of the printed construct. The thickness of the scanned layers is 890 µm. Scale bar in the phase contrast image is 200 µm. g) PrestoBlue assay to analyze the metabolic activity of the cells in the cell‐laden and cell‐seeded printed constructs over the course of 10 days of culture in vitro (*N* = 4, *p < 0.05, **p < 0.01). h,i) COLII staining and confocal imaging of soft ink layers on the edge of (h) and inside (i) the gel after 20 days of culture. Scale bar in the inset image is 50 µm. j) Percentage of collagen production per cell decreased slightly from the edge of the printed construct into the inner layers of the hydrogel. The fluorescence intensity of the confocal layers was measured and the ratio of this intensity to total numbers of DAPI gave out the percentage of collagen deposition per cell.

The 3D‐printed constructs composed of cell‐laden soft and stiff printed layers can be a suitable scaffold for seeding and encapsulation of chondrocytes to improve chondrogenic behavior in 3D and to promote the deposition of cartilage ECM. We first encapsulated the chondrocytes in the soft bioink and printed the multi‐material construct using the cell‐laden soft ink and acellular stiff ink in a dual nozzle printing process (**Figure** **4**a). Next, the cell‐laden constructs were allowed to stabilize and were cultured in vitro for 2 days. To enhance the cell viability and increase the cell density throughout the macroscale construct, additional amounts of chondrocytes were seeded on the printed constructs on day 2 of culture post printing. Seeding the chondrocytes on the printed constructs also allowed for higher cell signaling and interconnectivity across the construct. The printed constructs cultured with encapsulated cells sustained a high cell viability and adhesion to the biomaterial even after 30 days of culture in vitro (Figure 4b–d). The viability of encapsulated chondrocytes in the soft bioink was found to be approximately 80% on day 1 due to the loss of a fraction of cells to shear stress and the lack of cell culture conditions (Figure 4d). However, the cells recovered within 7 days of printing to an average viability of 95%. To perform the viability assay such as to be able to compare the values on day 1 and day 7 regardless of the effect of the seeding process, no cells were seeded in these constructs. Moreover, the encapsulated cells in the innermost layers of the constructs showed original rounded chondrocyte morphology (Figure S4a,b,d, Supporting Information) as opposed to the highly elongated morphology often observed in 2D cultures (Figure S4c, Supporting Information). Moving from the innermost to the outer layers of the constructs, the encapsulated cells gradually showed a relatively elongated morphology with a higher area of phalloidin (F‐actin) expression (Figure 4b,c). In terms of seeded cells on the printed constructs, Figure 4e,f shows the 3D reconstructed confocal images of cell morphologies cultured on the thick printed construct after 30 days. Accordingly, the cells covered the 3D construct along the printed fibers, creating a fully cellular macro‐scale viable construct. Moreover, the metabolic activity study of the cell‐laden printed and seeded constructs exhibited an increase over the course of 14 days (Figure 4g). The constructs were re‐plated after seeding and during culture to avoid media consumption by the excess cells migrating to the outside environment of the constructs and those attaching to the bottom of the plate. Therefore, the results of the metabolic assay were recorded merely based on the cells inside or on the constructs. Supplementing the chondrocytes with L‐ascorbic acid can promote COLII and GAGs deposition and lead to the effective differentiation of chondrocytes.^38^ Expression of COLII in the printed constructs supplemented with L‐ascorbic acid was observed after 14 days of culture (Figure 4h,i). The amount of collagen deposition decreased by moving from the edges of the constructs to inner sections (Figure 4j). This reduction can be pertained to the reduced delivery of L‐ascorbic acid and nutrients to the inner parts of the printed construct. In conclusion, the soft gel exhibited a supportive platform for 3D culture of chondrocytes.

### 2.3 Integration of the Biosensor and Hydrogel

**Figure 5:**
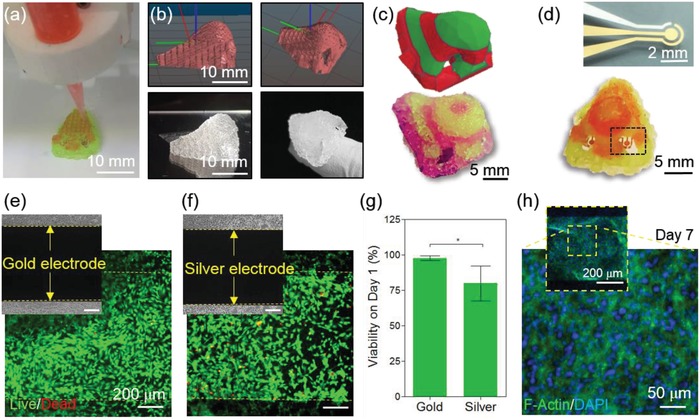
Integration of the biosensing system with the 3D‐printed construct. a) Dual ink nozzles printing a nose on top of the sensor electrodes. b,c) CAD 3D drawing of the nose with dual ink layers (top) and the printed construct using the same code (bottom). d) Optical image of the microfabricated Au biosensor with three electrode sensing system (top) and 3D‐printed dual‐ink nose integrated with the biosensors in each nostril (bottom). e,f) Live/dead assay images of chondrocytes seeded on the biosensor to test biocompatibility on Au (e) and Ag (f) electrodes. g) Quantified cell viability graph on Au and Ag electrodes on day 1 after integration with cells. h) F‐actin/DAPI staining of the sensor 7 days after integration.

The next step after optimization of the printing and chondrogenic maturation was creating the hybrid nose structure coupled with the sensing capability. As such, a CAD file of the nose was modified to include open nostril cavities for biosensor embedding (**Figure** **5**a–c). Printing the convex geometry of nose models along with nostril cavities required a relatively convoluted printing pattern with both convexities and concavities. The ink ratio of soft:stiff was therefore optimized to 1:2 to create a free‐standing nose to minimize the final structural (total layers: 10) collapse to less than 3% and to achieve geometrical integrity while maintaining porosity for nutrient perfusion as well as maintaining a chondrocyte‐supportive ECM through the soft layers. Next, the dual ink cell‐laden nose was printed on top of two functionalized sensors while the sensor chambers were left exposed (Figure 5a,d). To better illustrate the multi‐material printing of the nose using color contrasting, the nose in Figure 5c was printed using a 1:1 ink ratio (stiff layers shown in green and soft layers in red).

Interfacing tissues with electronics is still hampered by biocompatibility and cytotoxicity caused by the highly conductive but toxic elements used in the biosensing components, especially for the electrode materials and electrolytes. As such, it is essential to develop and optimize the biosensors in a cell‐friendly environment where the target biomimetic organ can remain viable while the functionality is maximized. Several groups have attempted to solve this issue by fabricating biocompatible electroconductive substrates such as graphene, carbon nanotubes, poly(3,4‐ethylenedioxythiophene):polystyrene sulfonate (PEDOT:PSS), and gold (Au) to provide a minimally toxicogenic environment for the integrated cell‐laden system.^39^ To achieve our aim, a metal‐based three‐microelectrode system with a working electrode (WE, Au), counter electrode (CE, Au), and reference electrode (RE, silver, Ag) was of specific interest for fabricating a robust EC biosensing system due to its decent stability, beneficial electron‐transfer kinetics, and capability to covalently bind with various chemical functional groups, namely, thiol‐based structures.^40^ In addition, to fabricate biocompatible microelectrodes, an e‐beam deposition method was selected to avoid using any toxic organic chemicals or compounds compared with other microfabrication methods. To evaluate the cytotoxicity of the electrode materials (Au and Ag), chondrocytes were seeded on the bare microelectrodes and their viability was analyzed. Using Au as a highly biocompatible conductive substrate, we observed 97.7 ± 1.3% cell viability (Figure 5e–g). The Ag RE showed decreased viability (79.7 ± 1.0%) compared with that of the Au electrode. However, the cytocompatibility of the Ag electrode was still good enough to allow for cell growth and proliferation after 7 days of culture as confirmed by F‐actin staining (Figure 5h).

### 2.4 Biosensing Explosive Molecules

**Figure 6:**
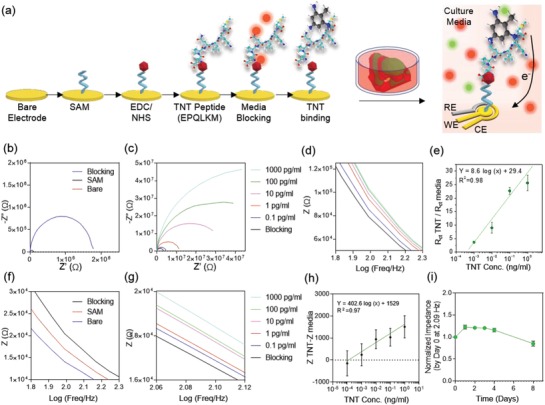
Biosensing mechanism and procedure. a) Schematic diagram of the functionalization procedure of the biosensor using the TNT‐specific peptide. b,c) Nyquist plot of measurement using different concentrations of TNT, sensing using ferrocyanide. Upon capturing TNT by the peptide, impedance increases in the ferrocyanide‐based measurement system. d) Bode plots using 0.1–1000 pg mL^−1^ TNT, sensing using ferrocyanide. Plots (c) and (d) share the same legend. e) Calibration curve for TNT sensing using ferrocyanide. f) Nyquist plots of measurement using 10 pg mL^−1^ of TNT, sensing using culture media. g) Bode plots using 0.1–1000 pg mL^−1^ TNT, sensing using culture media. h) Calibration curve for TNT sensing using cell culture media. i) Diagram of degradation of TNT peptide over 8 days of incubation at 37 °C.

EIS is a label‐free detection method which has recently been employed as a fast and reliable way to detect a myriad of chemical odors, airborne pathogens, and biomarkers with high sensitivity.^41^ Similar to the sensing mechanism in the olfactory epithelium, the EIS system can be functionalized to mimic the odor binding mechanism by creating a sensing platform similar to that performed through nasal mucus. We employed this detection method to develop a biosensing component for the hybrid nose (**Figure** **6**a). The method of fabrication and functionalization was developed and discussed in detail in a previously published paper where an Au‐based microelectrode was used as a highly conductive and effective substrate for label‐free monitoring of cell secretomes from in vitro cultured tissues.[qv: 41c]

Au‐based biosensing provides high flexibility in biosensor functionalization with a variety of natural or synthetic receptors to detect a wide range of chemical structures including explosives and human‐imperceptible biomolecules. Real‐time in situ detection of explosives is considered a major demand for security purposes and environmental concerns. Therefore, timely and sensitive detection of such chemicals can prevent a number of unfavorable and destructive events such as mine site blast injuries caused by explosive debris post explosion.^42^ 2,4,6‐Trinitrotoluene (TNT) is one of such prevalent explosives used for various industrial practices which can be captured and sensed using the EIS system. We tested the biosensing system with TNT using a TNT‐specific peptide chain (EPQLKM) developed in a previous paper.^43^ The mechanism of detection in the EIS‐based sensing system is premised on changes brought on by the interfacial electron transits between the redox probe [Fe(CN)_6_]^4−/3−^ and the substrate electrode upon creation of specific bonds between the antibody (here, TNT‐specific peptide) and the antigen (TNT).^44^ At a specific range of TNT concentrations, the amount of TNT captured by the receptor antibodies functionalized on the sensor corresponded to those present in the solution, and could be characterized via recording the electrode impedance.^44^ Therefore, we obtained the Nyquist curves in frequencies ranging from 10^−1^ to 10^5^ Hz (potential 0.10 V, and modulation amplitude, 5 mV) using a 50 × 10^−3^ m K_3_Fe(CN)_6_ electrolyte solution. As shown in Figure 6b, upon functionalization of a self‐assembled monolayer (SAM) and blocking media solution on the sensor, a significantly larger semicircle was seen in the high‐frequency region compared to the bare signal. Nonetheless, the signals recorded at low frequencies (i.e., Warburg impedance) were shown to be eliminated compared to the bare sensor signal. Subsequent functionalization of the sensor with analytes led to the binding of new molecules to the sensor surface, thus increasing the insulating layer between the probe and the electron transit. As such, the impedance at each functionalization step showed an increment compared to the former step (Figure 6b). Similarly, the diameter of the Nyquist semicircle (i.e., electron transfer resistance, *R*_ct_) at each step was also increased by immobilizing new layers. Next, the impedance was measured before and after the exposure of TNT molecules to the biosensor (Figure 6c,d). Similar to the receptor immobilization steps, exposing higher concentrations of TNT to the sensor led to the capture of more TNT molecules by the sensor and thus an increase in the recorded impedance and *R*_ct_ values. The results proved that the system can detect a range of 1–1000 pg mL^−1^ of TNT (Figure 6e). In addition, the biosensor exhibited capability to detect TNT with a limit of detection of 0.38 pg mL^−1^ with a sensitivity of 8.6 (log(ng mL^−1^))^−1^. Recently, Gao et al. implanted a TNT‐receptor functionalized bioelectronic nose sensor in the green fluorescent protein‐labeled olfactory sensory neurons (OSNs) of transgenic mice, showing detection sensitivity of up to 10 × 10^−6^ m TNT in vivo (11 ng mL^−1^).^45^

Potassium ferrocyanide (K_3_Fe(CN)_6_) is a common electrolyte mediator used for the EIS‐based measurements due to its strong redox activity.^46^ However, exposure of such electrolytes to cells and biological systems can cause cytotoxicity and lead to erroneous cell behavior.^18^ One solution to this problem is to seclude the measurement chamber from the incubation platforms to avoid direct cell contact with K_3_Fe(CN)_6 ._ 47 This solution, however, would not fit the purpose of a hybrid bionic nose which aims to monitor and measure the analytes in real‐time continual monitoring after being integrated with the engineered tissue. As an alternative, the biocompatibility of the system can be increased through changing the mediator electrolyte to a biocompatible nontoxic platform such as chondrocyte culture media (Figure 6f,g).^18^ As an initial step, we tested and compared the impedance response of the bare electrode to Dulbecco's phosphate buffer saline (PBS) and cell culture media, which contain ≈150 × 10^−3^ m salts and have an electrical conductivity of ≈1.5–2 S m^−1^ (Figure S5a,b, Supporting Information).^48^ However, in PBS and culture media, the semicircle curve disappeared due to the lack of or weakness of a redox reaction. The magnitude of the impedance recorded from the bare electrode in K_3_Fe(CN)_6_ was found to be 1000 times lower than PBS and culture media due to the relatively high resistance of media and PBS compared with that of K_3_Fe(CN)_6_. Next, the sensor was functionalized with an EPQLKM peptide and the results were measured in media and compared to those measured in the presence of K_3_Fe(CN)_6_ (Figure 6f–i). To compare the impedance (*Z*) values at any arbitrary frequency, such values can be extracted and drawn for different functionalization steps. As an example, we chose 2.09 Hz to compare the *Z* values of the sensors. As seen in Figure 6g, the impedance at 2.09 Hz follows an increasing trend upon increasing the concentration of TNT from 0.1 to 1000 pg mL^−1^. The calibration curve was calculated based on the different concentrations of TNT measured in media (Figure 6h). As a result, the sensitivity of the sensor was found to be 0.1 pg mL^−1^.

Considering that the developed hybrid system needs to be functional after days of culture at 37 °C, it is important to ensure that the functionalization of the sensor is not disturbed by the culture duration and environmental factors. Therefore, we performed a degradation test to confirm that the peptide can stay viably active in the culture environment for up to 8 days after printing and before being degraded (Figure 6i and Figure S5c, Supporting Information). The results proved that the peptide can stay active for up to 5 days after functionalization. Afterward, the gradual degradation of functionalized layers occurred, and the peptide coating layer was gradually destroyed from the substrate, leading to an impedance reduction to a value lower than the blocking stage. Therefore, for prolonged sensing, the sensor should be functionalized again or replaced by a new electrode after 5 days.

Consequently, the hybrid construct exhibited a potential to detect secretions of bioactive targets and disease biomarkers in humid mucus conditions. In later works, the detection mechanism can be fitted to provide biosensing in an integrated engineered mucus. Although the proposed approach introduces a different method for the integration of electronics with biomaterials, several challenges need to be addressed to reach a fully functional reconstructed nasal organ. For instance, embedding engineered co‐culture systems of neural and endothelial networks into the hybrid devices could be a first step toward compensating for the absence of vasculature and nerves in the thick printed constructs as opposed to the native organ.

Moreover, full retention of the human olfactory system requires the culture and integration of hundreds of ORs, combinations of which drive the smell recognition in the native olfactory epithelium.^49^ Although the emergence of the bioelectronic nose has pushed science toward artificial odor perception, there are still several challenges in reaching a full functional and implantable artificial nose. First, although the developed biosensors are capable of distinguishing their target odorants with high sensitivity and selectivity, they still fail to identify complex mixtures and reach a comprehensive odor perception system similar to that of humans.^50^ In fact, the natural olfactory‐taste system is stimulated by the combinatorial pattern recognition of various ORs,^49^, ^51^ and thus, the activation of several ORs would lead to the perception of a specific odor or taste. To reach higher levels of complex odor perception, the bionic nose devices require the implementation of a complete set of natural ORs which would jointly contribute to differentiation of diverse smells. Further, to enable odor discriminatory function in 3D bionic noses, it is essential to convert the single‐target biosensing system into a multisensory array functionalized with a range of ORs and peptide receptors. Recently, a multiplexed biosensor was proposed by Son et al.^50^ that immobilized human ORs onto a multichannel carbon nanotube‐based FET to analyze four types of taste‐ and odor‐causing compounds produced by food. Implementing such sensors in our hybrid system can generate a tunability to detect other targets such as a wide range of complex human‐perceptive odors using only a small OR matrix. In another study, an artificial multiplexed superbioelectronic nose was recently introduced by Kwon et al. to mimic the human odor discrimination in mixtures by functionalizing micro‐patterned graphene FETs with human ORs.[qv: 5h] Toward augmented detection of the bioelectronic nose through multiplex biosensing, Peveler et al. have developed a multichannel quantum dot array to detect five explosives including TNT up to 0.2 µg mL^−1^.^52^ Lastly, a crucial step toward biomimicking human olfaction is to standardize bionic nose devices by creating a universal code for gauging odors. This procedure can be done by choosing a set of primary odor molecules to build up odor mixtures and patterns, similar to the role of three primary light colors in building a wide spectrum of lights.^53^

Creation of hybrid bionic noses may contribute to understanding the fundamental mechanisms employed by the natural olfactory system for reaching selectivity. Eventually, the developed hybrid nose could promote a minimally invasive diagnosis tool for airway diseases, such as imminent asthmatic attacks, through sensing the airborne biomarkers in the breath. Moreover, 3D‐bioprinted hybrid nasal constructs could be instrumental in development of “cyborg” organs which possess augmented functionalities over their native human counterparts. Lastly, the developed hybrid system may offer enhanced precision to nasal cartilage reconstruction strategies as well as reviving the olfactory sensation which is often permanently lost during traumatic injuries. Therefore, the hybrid nasal construct can provide a potential solution to the drawback of current rhinectomy methods.

Being in its infancy, full nose organ retention still requires extensive studies and the addressing of several challenges. An artificial nose, which is to be implanted on the surface of the body, directly interacts with exogenous factors and the extrinsic environment, and thus cannot be fully maintained merely through the internal biological system for oxygen and nutrient supplementation, temperature regulation, and waste removal through blood circulation. A complementary external support is required to ensure the maintenance of environmental factors for tissue survival, or else the implant would be transformed into a massive necrotic block. To address this challenge, one solution is to add skin grafts on top of the artificial implant to isolate the organ from the outer environment and create uniform integration with the internal biosystem. This approach would require additional studies including the adhesion between the bionic nose implant and the skin graft.^54^ To obtain effective and accelerated integration with the host body on the initial days of implantation, engineered nose constructs must be supplemented with an array of pre‐vascularized networks which can connect with the host's vascular system. Additionally, another approach to integrate skin grafts could be suggested by co‐culturing the bionic nose with a skin‐mimetic layer (i.e., artificial skin)^55^ or 3D bioprinting a layer of tissue engineered skin on top of the bioprinted nose organ.^56^ In such a case, the bionic skin would potentially provide environmental necessities (e.g., temperature and humidity control, oxygen supply) for self‐maintaining the implant in the initial few weeks as well as releasing specific biological cues to induce skin regeneration. Finally, full replication of the anatomy and function of the nose will require reconstruction of the native structural and cellular heterogeneity including but not limited to cartilage, bone, OSNs, epithelial layers, and vasculature. This step would require concurrent advancement and research in multiple areas of tissue engineering including neural, skin, bone, and cartilage engineering.

## 3 Conclusion

We introduced a new hybrid device consisting of a dual bioink‐printed nose construct with an integrated biosensing system. The multi‐material printed construct consisted of several soft layers engineered for chondrogenic growth and adhesion, and multiple stiff layers with higher mechanical properties for promoting the mechanical robustness and macroscale geometrical integrity. The cell‐laden constructs supported chondrocyte growth and secretion of cartilage ECM after 14 days of culture in vitro. Moreover, the system showed capability in sensing TNT and could be further functionalized and tuned to detect a variety of natural odors, chemical structures, and disease biomarkers. The developed hybrid device lays the groundwork for a 3D‐printed viable cartilage‐like tissue with an integrated enhanced electronic olfactory system which can eventually become a viable humanoid cyborg nose organ. While these approaches can provide a more inclusive model of the native biological entities, they bring a myriad of new capabilities and novel applications in medicine, organ mimicry, and humanoid robotics by introducing controlled or even enhanced functionalities over the original biological functions. Lastly, future integration of a multiplex biosensing platform in the developed hybrid system and recapitulation of the multi‐layered heterogenous structure of the nasal cartilage can be a potential pathway toward achieving full nasal regeneration and nasal implants in the future.

## 4 Experimental Section

*Materials*: Gelatin from porcine skin, PEGDMA (1000 k), L‐ascorbic acid, and photo‐initiator (PI) 2‐hydroxy‐4′‐(2‐hydroxyethoxy)‐2‐methylpropiophenone (Irgacure D‐2959) were purchased from Sigma‐Aldrich. Hanks' balanced salt solution (HBSS), fetal bovine serum (FBS), PBS, and Dulbecco's modified Eagle medium (DMEM) were purchased from Thermo Fisher Scientific. Medium degree GelMA was synthesized based on the previous protocols.[qv: 25b] Briefly, 10% w/v gelatin was dissolved in PBS at 50 °C. Afterward, 5% v/v methacrylic anhydride (Sigma‐Aldrich) was added to the solution dropwise at a constant rate of 0.5 mL min^−1^. The reaction was mixed for 2 h. Next, PBS (40 °C) was added to the solution and the mixture was dialyzed (12–14 kDa dialysis membranes) at 40 °C for 7 days. The mixture was then placed at −80 °C for 2 days followed by lyophilization for 3 days. The resulting GelMA foam was stored at room temperature until use. SAM was prepared by dissolving 11‐mercaptoundecanoic acid (11‐MUA) (Sigma‐Aldrich) in >99% pure ethanol (Sigma‐Aldrich). *N*‐(3‐Dimethylaminopropyl)‐*N*'‐ethylcarbodiimide hydrochloride (EDC), *N*‐hydroxy‐succinimide 98% (NHS), and potassium ferricyanide [K_3_Fe(CN)_6_] were purchased from Sigma‐Aldrich. Ag epoxy glue (MG Chemicals) was used to connect the electrodes to the measurement system. A TNT‐specific peptide sequence (EPLQLKMGGGGWFVI) was purchased from Peptide 2.0. Peptide was diluted in deionized (DI) water at a concentration of 1 mg mL^−1^.

*Preparation of the Bioink*: To prepare the soft ink, 5% GelMA (medium degree of MA), 5% PEGDMA, 6.5% gelatin, and 0.5% PI were used. For the stiff ink, 8% GelMA (medium degree of MA), 10% PEGDMA, 3% gelatin, and 0.5% PI were used. To prepare the soft ink, PEGMDA was dissolved in HBSS and incubated at 80 °C for 20 min. Next, the GelMA, gelatin, and PI were added to the solution and the vial was covered by aluminum foil and incubated at 80 °C for 30 min. The ink was then moved to the 37 °C incubator for 20 min before being added to the cells. The preparation of stiff ink began by dissolving the gelatin in DI water and incubating at 80 °C for 10 min. Afterward, GelMA and PI were added to the ink, covered by aluminum foil and incubated at 80 °C for 20 min. Next, the PEGDMA was mixed with the solution and incubated at 37 °C for 30 min. The inks were allowed to stabilize at room temperature. Soft ink rested for 15 min prior to printing and Stiff ink required 40 min at room temperature before printing. Both times were coordinated to reach printable conditions at the same time.

*Mechanical Characterization*: Compression stress tests were performed using a parallel plate platform (ADMET, MTESTQuattro). Samples with around 0.9 cm circular diameter and 0.5 cm thickness were loaded and tested until rupture. All measurements were performed at room temperature. Prior to all measurements, the zero gap was determined. Four samples of each condition were tested. For the printed samples, an infill factor was introduced to normalize all the samples based on the theoretical (in the g‐code) and practical infill (after the print) by comparing the weights and strain of printed samples to bulk gels with the same dimensions. This infill factor was averaged and defined for the soft and stiff ink separately. For instance, to calculate the infill factor for 1:2, 1X soft and 2X stiff were used. All results of the composite ratios were normalized by their corresponding infill factors (0:1, 1:1, 1:2, 2:1, 1:0 and 0.89, 0.79, 0.75, 0.82, 0.68, respectively). To calculate Young's modulus, the elastic part (10–20% strain) of the stress–strain curve was used.

Rheology tests were performed on a TA Instruments DHR‐3 rheometer. A 20 mm diameter parallel plate geometry was used for all measurements. Samples were approximately 500 µm thick and the gap size was approximately 500 µm in all cases. All rheological measurements except the temperature sweep experiment were performed at 25 °C. To measure the viscosity by sweeping the temperature, a temperature control attachment (PolyScience) was used. Prior to measuring each sample, the measuring system inertia of the upper geometry as well as the motor friction was calibrated. All samples were allowed for relaxation and reached equilibrium for 30 min after loading and before initiating the test. An amplitude sweep was done on the samples to determine the linear viscoelastic window at three frequencies, ω = 0.3, 1, and 10 rad s^−1^, in 0.01–2% strain.

*3D Bioprinting*: 3D bioprinting was performed using a Cellink Inkredible bioprinter. A CAD file of a nose was sliced using Slic3r software to generate gcode readable by the bioprinter. The printing infill was chosen as 20% for both inks. A nozzle speed of 15 mm min^−1^ was used to print the final constructs. A 3 mL syringe (BD) and 27 gauge needle (Fisnar) were used for printing both inks. The nozzle was covered by foil during the process to avoid random crosslinking of the gel. An air pump was used to create the extrusion force for the nozzle. The pump rate was adjusted to initiate the printing and then decreased to 40 kPa for the soft ink and 90 kPa for the stiff ink. UV crosslinking was induced using a OmniCure S2000 machine and a 5 mm diameter light guide UV lamp. The stage was adjusted to 8 cm distance from the light guide and the UV intensity was calibrated to 100 mM cm^−2^. All constructs were crosslinked for 80 s, flipping the construct carefully half‐way through the process.

*Fabrication of the Sensors*: The sensors were fabricated at the Harvard Center for Nanoscale Systems. Ag RE, Au CE, and Au WE were created. After cleaning the square glass substrates (25 mm × 25 mm) with oxygen plasma, a shadow mask process was used in order to manufacture the microelectrodes (Figure S1, Supporting Information). In this process, metal layers were selectively deposited over a shadow mask which has apertures in a metal film of 0.25 mm thickness. In this process, the first shadow mask for WE and CE was attached to cleaned glass substrate. The 20 nm thick titanium (Ti), 20 nm thick palladium (Pd), and 500 nm thick Au were deposited on the glass using e‐beam evaporation. Also, the Au electrodes were not patterned with a passivation layer. Next, the second shadow mask for RE was attached to the Au deposited glass wafer. The alignment was achieved by using alignment keys. 20 nm thick Ti, 20 nm thick Pd, and 500 nm thick Ag were then deposited. After peeling off the shadow mask from the wafer, the required patterns were realized without the need for any wet processing.

*Sensor Functionalization to Detect TNT*: The functionalization of the sensor followed the previously established protocol.[qv: 41c] Briefly, the surface of the sensor was first functionalized with SAM using 11‐MUA. Next, an EDC/NHS conjugation was coated on the sensor to create a covalent bond between the surface and SAM where the carboxylterminated alkyl surface was converted to an active NHS ester when reacting with 11‐MUA. Next, the peptide was coated on the sensor at a concentration of 1 µg mL^−1^. Surface passivation was done using cell culture media. Dilutions of TNT were prepared in fresh cell culture media ranging from 0.1 pg mL^−1^ to 1.0 ng mL^−1^. The measurements were taken using K_3_Fe(CN)_6_ and cell culture media. In this case, the culture media was left at room temperature to reach pH equilibrium. Nyquist plots were obtained in the frequencies ranging from 10^−1^ to 10^5^ Hz under a potential value of 0.1 V and modulation amplitude of 5.0 mV in 50 × 10^−3^ m K_3_Fe(CN)^6^.

*Toxicity Test of the Biosensor*: All sensors were washed with 70% ethanol followed by 30 min incubation at 37 °C with 1% Anti‐Anti in PBS solution.

*In Vitro Culture*: Primary chondrocytes from 1 month old calves (Astarte bio) were cultured and passaged for 1 week at 37 °C and 5% CO_2_. DMEM, 10% FBS, and 1% p/s were used as the culture media. Cells were encapsulated in the bioink at a density of 20 million cells mL^−1^. Furthermore, additional cells with 0.5 million cells cm^−2^ were seeded on the constructs on day 2 of printing. After printing, the culture media was supplemented with 50 µg mL^−1^ l‐ascorbic acid to induce collagen II production. To increase the cell viability during the printing process, the concentration of HBSS in the soft ink was adjusted to include 20% FBS added directly to the cell pellet before cell encapsulation (1:9 ratio of HBSS to FBS). The constructs were supplemented with media after printing and the media were changed every 2 days.

*Cell‐Laden Characterization*: Cell viability and proliferation of the printed constructs were assessed using live/dead assay (Thermo Fisher scientific) and PrestoBlue kit (Thermo Fisher scientific). The colorimetric assays were measured using a plate reader (Infinite 200 pro, Tecan Austria GmbH) by measuring the absorbance at 570 nm with reference to 600 nm. The results were normalized by day 1 of culture. Fixation of cell‐laden constructs was done on days 14 and 30. Briefly, the constructs were treated with 4% paraformaldehyde solution (Thermo Fisher scientific) for 30 min and then permeabilized using 0.1% Triton X (Sigma‐Aldrich). F‐actin (1:40, Invitrogen) and COLII antibody (1:100, Invitrogen) were added to the constructs for staining. The constructs were incubated with the primary antibody overnight on a mild shaker at 4 °C. Secondary antibody goat anti‐mouse Alexa Fluor 594 (Invitrogen) was used to image the COLII staining and was added to the constructs, followed by incubation at 4 °C for 6 h. DAPI (4′,6‐diamidino‐2‐phenylindole, 1:1000, Sigma‐Aldrich) was added 30 min prior to imaging. Confocal imaging was performed using a Zeiss LSM 880 airyscan microscope. Images were processed and analyzed in Fiji software.

*Statistical Analysis*: All tests were performed in triplicates and the average and standard deviation were calculated in Graphpad Prism. The results of the PrestoBlue assay were analyzed using one‐way analysis of variance method. Error bars represented mean ± standard deviation of measurements in each group. To compare different treatment groups and study the existence of significant differences among groups, Tukey's multiple comparison method with *p* < 0.05 was employed. Statistical Significance in all graphs was indicated as not significant (ns) (*p* > 0.0.5), *(*p* < 0.04), and ****(*p* < 0.0001).

## Conflict of Interest

The authors declare no conflict of interest.

## Supporting information

Supplementary information

Supplemental Movie 1

## References

[1] J. V. Pagaduan , A. Bhatta , L. H. Romer , D. H. Gracias , Small 2018, 14, 1702497. 10.1002/smll.201702497 29749014

[2] a) F. Pati , J. Gantelius , H. A. Svahn , Angew. Chem., Int. Ed. 2016, 55, 4650; 10.1002/anie.201505062 26895542

[3] M. R. Homicz , K. B. McGowan , L. M. Lottman , G. Beh , R. L. Sah , D. Watson , Arch. Facial Plast. Surg. 2003, 5, 53. 12533140 10.1001/archfaci.5.1.53

[4] O. Bas , E. M. De‐Juan‐Pardo , C. Meinert , D. D'Angella , J. G. Baldwin , L. J. Bray , R. M. Wellard , S. Kollmannsberger , E. Rank , C. Werner , Biofabrication 2017, 9, 025014. 28374682 10.1088/1758-5090/aa6b15

[5] a) T. H. Kim , S. H. Lee , J. Lee , H. S. Song , E. H. Oh , T. H. Park , S. Hong , Adv. Mater. 2009, 21, 91;

[6] W. Hu , L. Wan , Y. Jian , C. Ren , K. Jin , X. Su , X. Bai , H. Haick , M. Yao , W. Wu , Adv. Mater. Technol. 2019, 4, 1800488.

[7] S. Zampolli , I. Elmi , F. Ahmed , M. Passini , G. Cardinali , S. Nicoletti , L. Dori , Sens. Actuators, B 2004, 101, 39.

[8] J. Gruber , H. M. Nascimento , E. Y. Yamauchi , R. W. Li , C. H. Esteves , G. P. Rehder , C. C. Gaylarde , M. A. Shirakawa , Mater. Sci. Eng., C 2013, 33, 2766. 10.1016/j.msec.2013.02.043 23623094

[9] C. W. Hanson , E. R. Thaler , Anesthesiology 2005, 102, 63. 15618788 10.1097/00000542-200501000-00013

[10] J.‐E. Chang , D.‐S. Lee , S.‐W. Ban , J. Oh , M. Y. Jung , S.‐H. Kim , S. Park , K. Persaud , S. Jheon , Sens. Actuators, B 2018, 255, 800.

[11] a) K. Feron , R. Lim , C. Sherwood , A. Keynes , A. Brichta , P. Dastoor , Int. J. Mol. Sci. 2018, 19, 2382; 10.3390/ijms19082382 PMC6121695 30104515

[12] a) L. Lavernia , W. E. Brown , B. J. Wong , J. C. Hu , K. A. Athanasiou , Acta Biomater. 2019, 88, 42; 30794988 10.1016/j.actbio.2019.02.025

[13] a) E. Prokopakis , M. Doulaptsi , A. Karatzanis , H. Kawauchi , Turk Arch Otorhinolaryngol 2019, 57, 39; 31049252 10.5152/tao.2019.3889 PMC6461332

[14] a) J. R. Harkema , S. A. Carey , J. G. Wagner , Toxicol. Pathol. 2006, 34, 252; 16698724 10.1080/01926230600713475

[15] Z. Chang , Y. Sun , Y. Zhang , Y. Gao , X. Weng , D. Chen , L. David , J. Xie , J. Bionic Eng. 2018, 15, 533.

[16] M. S. Mannoor , Z. Jiang , T. James , Y. L. Kong , K. A. Malatesta , W. O. Soboyejo , N. Verma , D. H. Gracias , M. C. McAlpine , Nano Lett. 2013, 13, 2634. 23635097 10.1021/nl4007744 PMC3925752

[17] a) A. D. Valentine , T. A. Busbee , J. W. Boley , J. R. Raney , A. Chortos , A. Kotikian , J. D. Berrigan , M. F. Durstock , J. A. Lewis , Adv. Mater. 2017, 29, 1703817; 10.1002/adma.201703817 28875572

[18] J. Lee , S. Shin , A. Desalvo , G. Lee , J. Y. Lee , A. Polini , S. Chae , H. Jeong , J. Kim , H. Choi , Adv. Healthcare Mater. 2017, 6, 1700231. 10.1002/adhm.201700231 28636127

[19] a) P. Reich , R. Stoltenburg , B. Strehlitz , D. Frense , D. Beckmann , Int. J. Mol. Sci. 2017, 18, 2484; 10.3390/ijms18112484 PMC5713450 29160851

[20] A. Wilson , Metabolites 2015, 5, 140.25738426

[21] R. Genç , in Nanobiosensors (Ed: Grumezescu A. M.), Academic Press/Elsevier, 2017, ch. 10, p. 391.

[22] J. Leppiniemi , P. Lahtinen , A. Paajanen , R. Mahlberg , S. Metsä‐Kortelainen , T. Pinomaa , H. Pajari , I. Vikholm‐Lundin , P. Pursula , V. P. Hytönen , ACS Appl. Mater. Interfaces 2017, 9, 21959. 28598154 10.1021/acsami.7b02756

[23] Z. Zheng , J. Wu , M. Liu , H. Wang , C. Li , M. J. Rodriguez , G. Li , X. Wang , D. L. Kaplan , Adv. Healthcare Mater. 2018, 7, 1701026. 10.1002/adhm.201701026 29292585

[24] a) P. Shi , A. Laude , W. Y. Yeong , J. Biomed. Mater. Res., Part A 2017, 105, 1009; 10.1002/jbm.a.35971 27935198

[25] a) I. Noshadi , S. Hong , K. E. Sullivan , E. S. Sani , R. Portillo‐Lara , A. Tamayol , S. R. Shin , A. E. Gao , W. L. Stoppel , L. D. Black III , Biomater. Sci. 2017, 5, 2093; 28805830 10.1039/c7bm00110j PMC5614854

[26] T. Billiet , E. Gevaert , T. De Schryver , M. Cornelissen , P. Dubruel , Biomaterials 2014, 35, 49. 24112804 10.1016/j.biomaterials.2013.09.078

[27] J. D. Richmon , A. Sage , V. W. Wong , A. C. Chen , R. L. Sah , D. Watson , Am. J. Rhinol. 2006, 20, 496. 17063745 10.2500/ajr.2006.20.2932

[28] P. A. Levett , F. P. Melchels , K. Schrobback , D. W. Hutmacher , J. Malda , T. J. Klein , Acta Biomater. 2014, 10, 214. 24140603 10.1016/j.actbio.2013.10.005

[29] I. E. Erickson , S. R. Kestle , K. H. Zellars , M. J. Farrell , M. Kim , J. A. Burdick , R. L. Mauck , Acta Biomater. 2012, 8, 3027. 22546516 10.1016/j.actbio.2012.04.033 PMC3389207

[30] E. van den Bosch , C. Gielens , Int. J. Biol. Macromol. 2003, 32, 129. 12957309 10.1016/s0141-8130(03)00046-1

[31] C. B. Hutson , J. W. Nichol , H. Aubin , H. Bae , S. Yamanlar , S. Al‐Haque , S. T. Koshy , A. Khademhosseini , Tissue Eng., Part A 2011, 17, 1713. 21306293 10.1089/ten.tea.2010.0666 PMC3118706

[32] M. M. Caron , P. J. Emans , M. M. Coolsen , L. Voss , D. A. Surtel , A. Cremers , L. W. van Rhijn , T. J. Welting , Osteoarthritis Cartilage 2012, 20, 1170. 22796508 10.1016/j.joca.2012.06.016

[33] M. B. Goldring , in Human Cell Culture Protocols (Ed: Picot J.), Humana Press, Totowa, NJ, USA 2005, p. 69.

[34] P. D. Benya , J. D. Shaffer , Cell 1982, 30, 215. 7127471 10.1016/0092-8674(82)90027-7

[35] H. Hauselmann , R. J. Fernandes , S. S. Mok , T. M. Schmid , J. A. Block , M. B. Aydelotte , K. E. Kuettner , E. Thonar , J. Cell Sci. 1994, 107, 17. 8175906 10.1242/jcs.107.1.17

[36] G. J. Gibson , S. L. Schor , M. E. Grant , J. Cell Biol. 1982, 93, 767. 6811598 10.1083/jcb.93.3.767 PMC2112156

[37] J. W. Nichol , S. T. Koshy , H. Bae , C. M. Hwang , S. Yamanlar , A. Khademhosseini , Biomaterials 2010, 31, 5536. 20417964 10.1016/j.biomaterials.2010.03.064 PMC2878615

[38] S. Pahoff , C. Meinert , O. Bas , L. Nguyen , T. J. Klein , D. W. Hutmacher , J. Mater. Chem. B 2019, 7, 1761. 10.1039/c8tb02607f 32254918

[39] a) S. R. Shin , B. Migliori , B. Miccoli , Y. C. Li , P. Mostafalu , J. Seo , S. Mandla , A. Enrico , S. Antona , R. Sabarish , Adv. Mater. 2018, 30, 1704189; 10.1002/adma.201704189 PMC6082116 29323433

[40] a) S. C. Kwok , F. Ciucci , M. M. Yuen , Electrochim. Acta 2016, 198, 185;

[41] a) R. Khadka , N. Aydemir , C. Carraher , C. Hamiaux , D. Colbert , J. Cheema , J. Malmström , A. Kralicek , J. Travas‐Sejdic , Biosens. Bioelectron. 2019, 126, 207; 30415156 10.1016/j.bios.2018.10.043

[42] J. Wang , Electroanalysis 2007, 19, 415.

[43] Y. Cui , S. N. Kim , S. E. Jones , L. L. Wissler , R. R. Naik , M. C. McAlpine , Nano Lett. 2010, 10, 4559. 20942387 10.1021/nl102564d

[44] L. Pires Carneiro , Development of an Electrochemical Biosensor Platform and a Suitable Low‐Impedance Surface Modification Strategy, KIT Scientific Publishing, Karlsruhe, Germany 2014.

[45] K. Gao , S. Li , L. Zhuang , Z. Qin , B. Zhang , L. Huang , P. Wang , Biosens. Bioelectron. 2018, 102, 150. 29128717 10.1016/j.bios.2017.08.055

[46] a) S. Vogt , Q. Su , C. Gutiérrez‐Sánchez , G. Nöll , Anal. Chem. 2016, 88, 4383; 26990929 10.1021/acs.analchem.5b04814

[47] a) P. Bombelli , T. Müller , T. W. Herling , C. J. Howe , T. P. Knowles , Adv. Energy Mater. 2015, 5, 1401299; 10.1002/aenm.201401299 PMC4503997 26190957

[48] A. Mazzoleni , B. Sisken , R. Kahler , Bioelectromagnetics 1986, 7, 95. 3730006 10.1002/bem.2250070111

[49] S. Firestein , Nature 2001, 413, 211. 11557990 10.1038/35093026

[50] M. Son , D. Kim , H. J. Ko , S. Hong , T. H. Park , Biosens. Bioelectron. 2017, 87, 901. 27664409 10.1016/j.bios.2016.09.040

[51] a) H. Saito , Q. Chi , H. Zhuang , H. Matsunami , J. D. Mainland , Sci. Signaling 2009, 2, ra9; 10.1126/scisignal.2000016 PMC2774247 19261596

[52] W. J. Peveler , A. Roldan , N. Hollingsworth , M. J. Porter , I. P. Parkin , ACS Nano 2016, 10, 1139. 26579950 10.1021/acsnano.5b06433

[53] M. Son , J. Y. Lee , H. J. Ko , T. H. Park , Trends Biotechnol. 2017, 35, 301. 28089199 10.1016/j.tibtech.2016.12.007

[54] K. Vig , A. Chaudhari , S. Tripathi , S. Dixit , R. Sahu , S. Pillai , V. A. Dennis , S. R. Singh , Int. J. Mol. Sci. 2017, 18, 789. 10.3390/ijms18040789 PMC5412373 28387714

[55] X. Pan , Q. Wang , P. He , K. Liu , Y. Ni , L. Chen , X. Ouyang , L. Huang , H. Wang , S. Xu , Chem. Eng. J. 2020, 379, 122271.

[56] R. Augustine , Prog. Biomater. 2018, 7, 77. 29754201 10.1007/s40204-018-0087-0 PMC6068049

