# Supplementary material for: A 3D‐Printed Hybrid Nasal Cartilage with Functional Electronic Olfaction
Source: Adv Sci (Weinh). 2020 Jan 10;7(5):1901878. doi: 10.1002/advs.201901878 (PMC7055567; doi:10.1002/advs.201901878)
Supplement: Supplementary file 1 — Supplementary information [file ADVS-7-1901878-s001.pdf]

Copyright WILEY-VCH Verlag GmbH & Co. KGaA, 69469 Weinheim, Germany, 2019.

ADVANCED SCIENCE
Open Access

Supporting Information

for *Adv. Sci.,* DOI: 10.1002/advs.201901878

A 3D-Printed Hybrid Nasal Cartilage with Functional Electronic Olfaction

Yasamin A. Jodat, Kiavash Kiaee, Daniel Vela Jarquin, Rosakaren Ludivina De la Garza Hernández, Ting Wang, Sudeep Joshi, Zahra Rezaei, Bruna Alice Gomesde Melo, David Ge, Manu S. Mannoor, and Su Ryon Shin**

DOI:
Article type: Full Paper

A 3D Printed Hybrid Nasal Cartilage with Functional Electronic Olfaction

Yasamin A. Jodat, Kiavash Kiaee, Daniel Vela Jarquin, Rosakaren Ludivina De la Garza Hernández, Ting Wang, Sudeep Joshi, Zahra Rezaei, Bruna Alice Gomes de Melo, David Ge, Manu S. Mannoor, Su Ryon Shin*.*

Y. A. Jodat, K. Kiaee, D. V. Jarquin, L.R. De la Garza-Hernández, T. Wang, Z. Rezaei, B. A. G. de Melo, D. Ge, Dr. S. R. Shin.
Division of Engineering in Medicine, Department of Medicine, Harvard Medical School, Brigham and Women's Hospital, Cambridge, MA 02139, USA.
*E-mail: sshin4@bwh.harvard.edu

Y. A. Jodat, K. Kiaee, Dr. S. Joshi, Prof. M. S. Mannoor
Department of Mechanical Engineering, Stevens Institute of Technology, Hoboken, NJ, 07030, USA.
*E-mail: mmannoor@stevens.edu

D. V. Jarquin
Instituto Tecnológico y de Estudios Superiores de Monterrey, Calle del Puente #222 Col. Ejidos de Huipulco, Tlalpan C.P. 14380, México D.F.

L.R. De la Garza-Hernández
Instituto Tecnológico y de Estudios Superiores de Monterrey, Av. Eugenio Garza Sada 2501 Sur, Tecnológico, 64849 Monterrey, N.L., Mexico

T. Wang
School of Medicine, Jiangsu University, Zhenjiang, Jiangsu 212013, China.

Z. Rezaei, Department of Chemical and Petroleum Engineering, Sharif University of Technology, Azadi Ave, 11365-11155, Tehran, Iran

B. A. G. de Melo
Department of Engineering of Materials and Bioprocesses, School of Chemical Engineering, University of Campinas, Campinas, SP 13083-852, Brazil.

[*] S.R. Shin and M.S Mannoor contributed equally as corresponding authors.

Keywords: Biomaterials, 3D bioprinting, Electrochemical biosensors, Bioelectronic noses, Bionic organs

Table S1. Rheological parameters obtained from the power law model for soft and stiff bioinks.

**Bioink**	***K* (Pa.s^n)**	***N***
**Soft**	10.08	0.458
**Stiff**	12.56	0.746

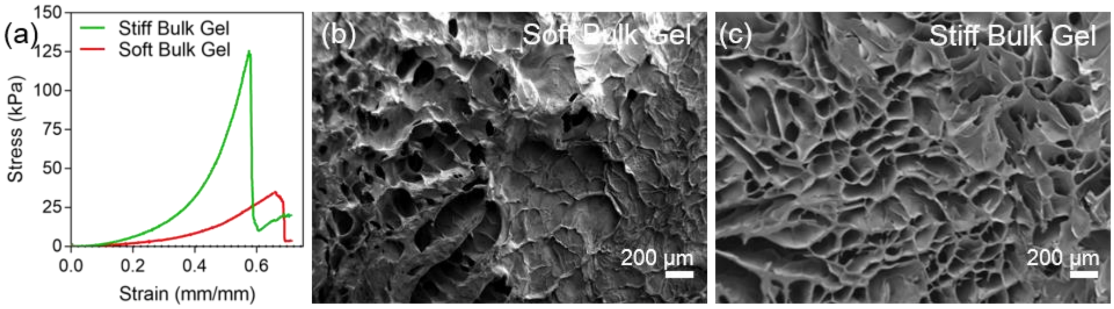

Figure S1. a) Stress strain curves of soft (red) and stiff (green) bulk gels (non-printed) up to the fracture point. b) and c) SEM imaging of bulk gels. Soft gel collapses while stiff gel maintains the structure micro-porosity.

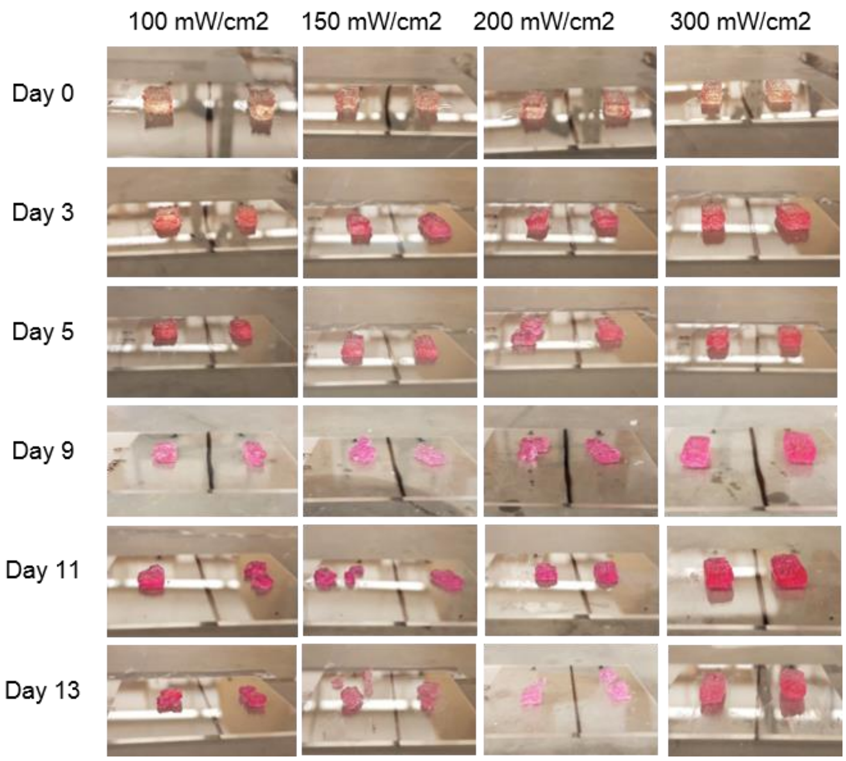

Figure S2. Tunable degradation by adjusting the UV crosslinking intensity.

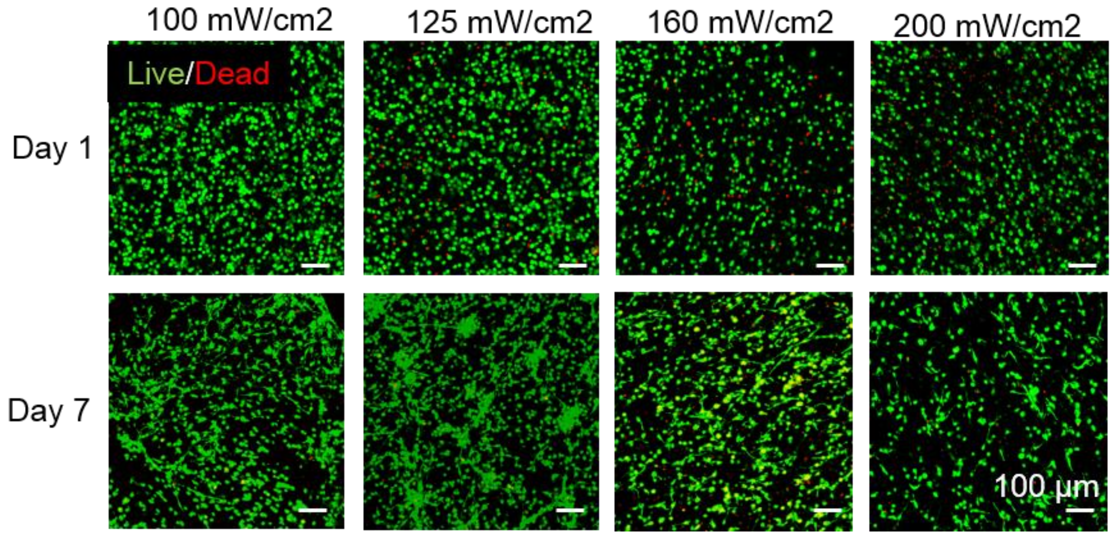

Figure S3. Effect of UV on cell proliferation and morphology. Cells in differently crosslinked soft gels were live stained using Live/Dead staining (green: live, red: dead)

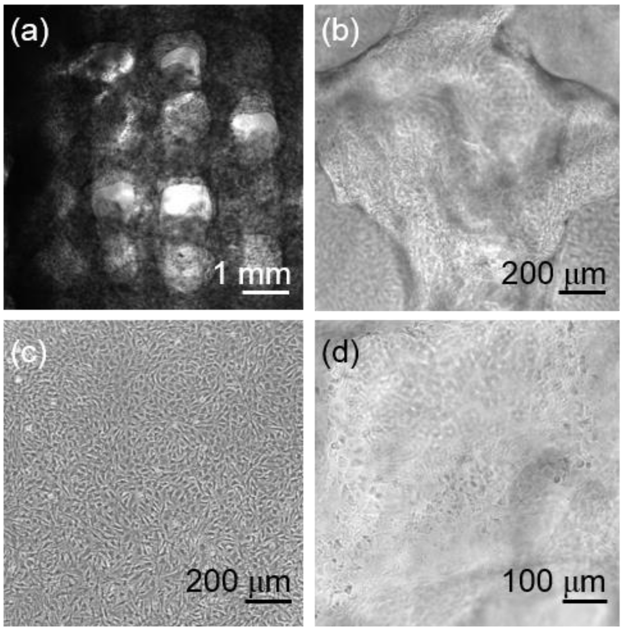

Figure S4. Phase contrast imaging of printed constructs. a) Low magnification of the cell-laden printed grid. b) and d) The cells maintain a round morphology inside the 3D gel in contrast to the dedifferentiated cells in the 2D wellplate (c).

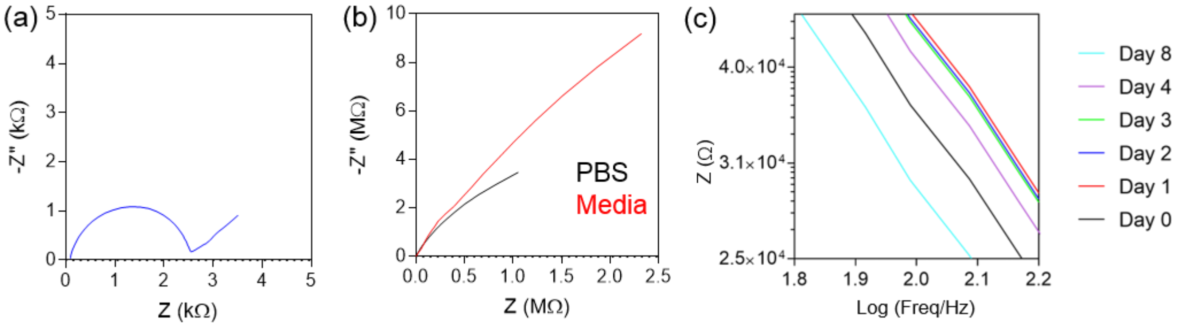

Figure S5. a) and b) Nyquist plots of bare sensor signal tested in $K_3Fe(CN)_6$, PBS and Media. Comparison of bare signals achieved from cell culture media, $K_3Fe(CN)_6$ and PBS used as electrolytes. c) Peptide degradation assay, signal recording from the sensor after incubation at 37 °C for eight days.

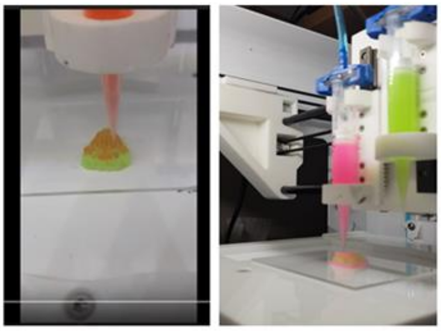

Video S1. Video of printing the nose using the dual printing system.
